# Acute coronary syndrome after liver transplantation in a young primary biliary cholangitis recipient with dyslipidemia: a case report

**DOI:** 10.1186/s40792-022-01470-1

**Published:** 2022-06-17

**Authors:** Siyuan Yao, Akiho Iwashita, Shintaro Yagi, Hirotoshi Watanabe, Takahiro Nishio, Yukinori Koyama, Kazuyuki Nagai, Naoko Kamo, Kojiro Taura, Etsuro Hatano

**Affiliations:** 1 Department of Surgery, Graduate School of Medicine, Kyoto University, Kyoto, Japan; 2 Department of Surgery, Kobe City Medical Center West Hospital, Hyogo, Japan; 3 Department of Surgery, Graduate School of Medicine, Kanazawa University, Ishikawa, Japan; 4 Department of Cardiovascular Medicine, Graduate School of Medicine, Kyoto University, Kyoto, Japan

**Keywords:** Primary biliary cholangitis, Liver transplantation, Hypercholesterolemia, Hypertriglyceridemia, Coronary artery disease

## Abstract

**Background:**

Primary biliary cholangitis (PBC) is a chronic, progressive liver disease associated with dyslipidemia. There is a consensus that PBC does not accelerate coronary artery disease despite high cholesterol levels, so the screening test for the coronary artery is not routinely performed before liver transplantation (LT). To date, no report has described the potential risk of PBC-related dyslipidemia for developing acute coronary syndrome (ACS) after LT.

**Case presentation:**

A 40-year-old Asian female with a known history of PBC underwent ABO-incompatible living-donor LT, with her husband as the donor. Although she had high cholesterol and triglyceride levels that were refractory to medications, she passed all routine preoperative examinations, including cardiopulmonary function tests and infection screenings. One week after LT, she developed ACS with 90% stenosis of both the left anterior descending artery and left circumflex artery. Emergent stent implantation was successfully performed followed by dual antiplatelet therapy. The long history of PBC and associated severe dyslipidemia for 10 years would have accelerated the atherosclerosis, causing latent stenosis in the coronary artery. Inapparent stenosis might have become apparent due to unstable hemodynamics during the acute phase after LT.

**Conclusions:**

PBC-related dyslipidemia potentially brings a risk for developing ACS after LT. This experience suggests that the preoperative evaluation of the coronary artery should be considered for high-risk patients, especially those who have drug-resistant dyslipidemia.

## Introduction

Primary biliary cholangitis (PBC) is a chronic, progressive liver disease associated with markedly elevated serum lipids. PBC is characterized by the loss of interlobular bile ducts due to degeneration and necrosis of bile duct epithelial cells, resulting in cholestasis and dyslipidemia. Since previous large observational studies failed to find an increased risk of serious coronary artery disease (CAD) in PBC patients, there is a consensus that PBC does not accelerate CAD despite high cholesterol levels [1, 2]. The practice guidelines for PBC do not cite the necessity of coronary artery investigation [3, 4]; thus, screening tests of the coronary artery are not routinely performed before liver transplantation (LT) at our department except for electrocardiogram and echocardiogram. Herein, we describe a young female patient with PBC-associated dyslipidemia who developed acute coronary syndrome (ACS) in the acute phase after living-donor liver transplantation (LDLT). This was the first case of ACS after LDLT among 1,980 recipients, including 125 with PBC, in the 30-year history of LT in our institution. The aim of this report is to demonstrate its unusual presentation and discuss the appropriate management strategy.

## Case presentation

**Fig. 1:**
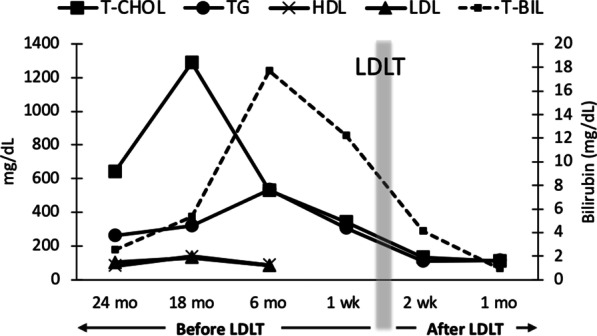
Chronological changes in parameters. *LDLT* living donor liver transplantation, *T-CHOL* total cholesterol, *TG* triglycerides, *HDL* high-density lipoprotein, *LDL* low-density lipoprotein, *T-BIL* total bilirubin

A 40-year-old Asian female with a known history of PBC underwent an ABO-incompatible LDLT (B to O), with her 31-year-old husband as the living donor. The patient was diagnosed with PBC at the age of 30 and was under treatment with ursodeoxycholic acid (UDCA) and pemafibrate for cholestasis and dyslipidemia. Despite treatment, her cholestasis worsened over time. The results of laboratory tests before LT are shown in Table 1. The Child–Pugh score, the Model for End-Stage Liver Disease score and the Mayo PBC risk score were 9, 12 and 9.98, respectively. She passed all routine preoperative examinations, including cardiopulmonary function tests (chest X-ray, spirometry, electrocardiogram, and echocardiography) and infection screenings. Despite the medication, she had a total cholesterol level of 343 mg/dL (normal range 140–219) and a triglyceride level of 307 mg/dL (normal range 38–149). Both high-density lipoprotein cholesterol and low-density lipoprotein (LDL) cholesterol were within the normal ranges. The chronological changes in these parameters and bilirubin are presented in Fig. 1. On physical examination, typical multiple palpebral and palmar xanthomas were observed. She had no other cardiovascular risk factors, such as smoking history, hypertension, diabetes mellitus, or family history of CAD. Her body mass index was 20.1 kg/m^2^. Contrast-enhanced computed tomography (CT) did not show atherosclerotic plaques or calcifications in coronary arteries and abdominal vessels.

**Table 1:** Laboratory data before transplantation

Pre-LT laboratory data					
WBC	5600	/μL	Creatinine	0.61	mg/dL
Hemoglobin	8.5	g/dL	eGFR	85.4	mL/min/1.73 m^2^
Platelet	17.7	× 10^4^/μL	Total cholesterol	343	mg/dL
INR	1.12		Triglyceride	307	mg/dL
AST	169	U/L	HDL	87	mg/dL
ALT	55	U/L	LDL	85	mg/dL
ALP	1772	U/L	Ammonia	49	μg/dL
γ-GTP	397	U/L	CRP	6.9	mg/dL
Albumin	2.6	g/dL	Child–Pugh score	9	
Total bilirubin	12.5	mg/dL	MELD score	12	
Direct bilirubin	8.7	mg/dL	Mayo risk score	9.98	

*LT* liver transplantation, *WBC* white blood cell, *INR* international normalized ratio, *AST* aspartate aminotransferase, *ALT* alanine aminotransferase, *ALP* alkaline phosphatase, *γ-GTP* γ-glutamyl transpeptidase, *GFR* estimated glomerular filtration rate, *HDL* high-density lipoprotein, *LDL* low-density lipoprotein, *CRP* C-reactive protein, *MELD* model for end-stage liver disease

LDLT using a left-lobe graft was performed following rituximab induction and went without any serious intraoperative complications. Simultaneous splenectomy was performed for a low graft-to-spleen volume ratio based on our institutional strategy [5]. The operative time was 616 min, and blood loss was 1390 mL. Transfused red blood cells, fresh frozen plasma, and platelets were 560 mL, 240 mL, and 200 mL, respectively. The graft-to-recipient weight was 0.63%. The pathological findings of the original liver were compatible with PBC, and there was no evidence of malignancy.

**Fig. 2:**
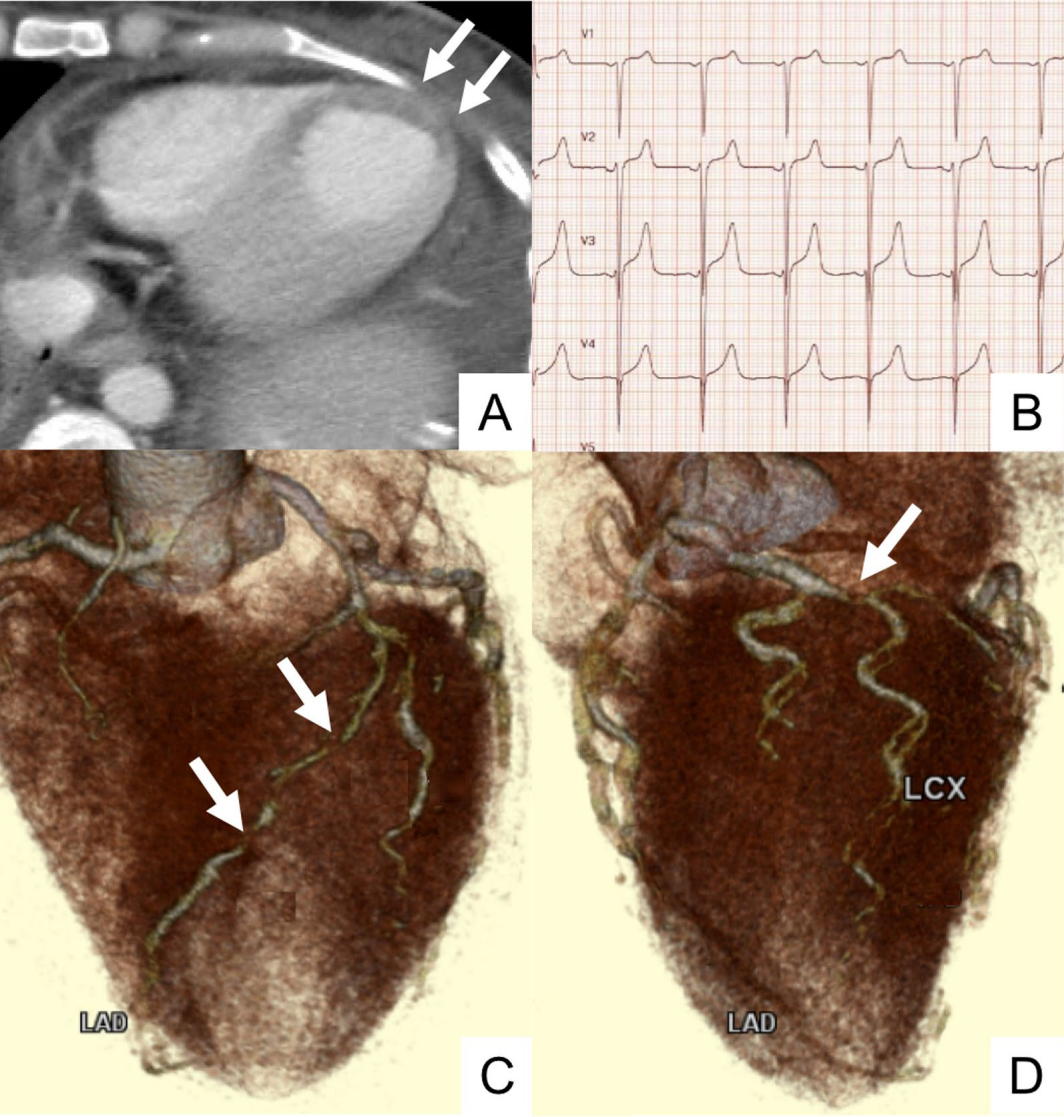
Cardiovascular evaluation. **A** Poor contrast enhancement in the apex (white arrow) on CT on POD 7. **B** ST elevation in leads V1–4 on the electrocardiogram on POD 9. **C** Suspicious stenosis in the left anterior descending artery on coronary CT angiography on POD 13 (white arrow). **D** Suspicious stenosis in the left circumflex artery on coronary CT angiography on POD 13 (white arrow). *AV* AV node artery, *CT* computed tomography, *LAD* left anterior descending artery, *LCX* left circumflex artery, *PD* poster descending branch, *POD* postoperative day

**Fig. 3:**
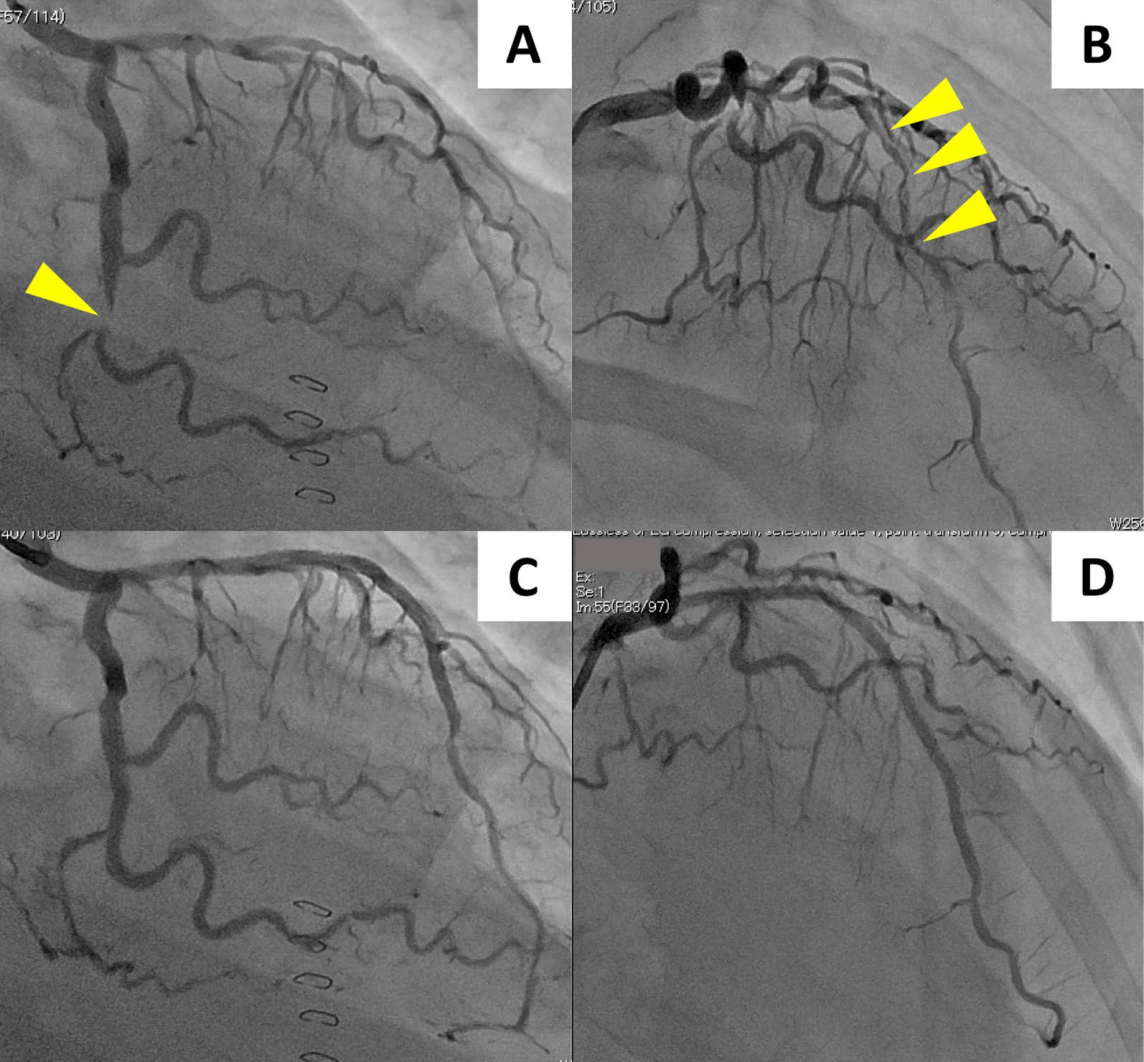
Coronary angiography. **A** Stenosis of the left anterior descending artery (yellow arrow). **B** Stenosis of the left circumflex artery (yellow arrows). **C** Successful stent placement in the left anterior descending artery. **D** Successful stent placement in the left circumflex artery

The postoperative course was uneventful until POD 7, when hypotension emerged. She had never complained about chest pain before. Routine procedures, including cultures and contrast-enhanced CT, showed no evidence of infection. Since liver biopsy on POD 7 proved cellular rejection, steroid pulse therapy was immediately started. However, despite intense administration of catecholamine, the hypotension was refractory. On POD 9, poor contrast enhancement in the apex of the heart was noted by the radiologist’s review of the prior CT (Fig. 2A), and consequent electrocardiogram showed ST elevation in leads V1–4 (Fig. 2B). Troponin T and creatinine kinase MB (CK-MB) were elevated to 1.050 ng/mL (normal range ≤ 0.014) and 35 U/L (normal range ≤ 25), respectively. The patient was first referred to the cardiologist. As her echocardiogram showed akinesia of the apex and contrastive hypercontraction at the base, the diagnosis of Takotsubo cardiomyopathy was made. Although conservative treatment by fluid loading and vasopressor administration was continued, the response was poor, and symptoms such as nausea and chest pain appeared. On POD 13, coronary CT angiography (CCTA) was performed for further evaluation, and stenoses of the left anterior descending artery (Fig. 2C) and the left circumflex artery (Fig. 2D) were suspected. Troponin T was elevated to 2.750 ng/mL, and CK-MB was 33 U/L. The diagnosis of ACS was made, and emergent percutaneous coronary intervention was performed on POD 13. Emergent coronary angiography demonstrated 90% stenosis of both the left anterior descending artery (#6–7) and the left circumflex artery (#14) (Fig. 3A, B). Two drug-eluting stents (DESs) in the left anterior descending artery and one DES in the left circumflex artery were implanted (Fig. 3C, D). Dual antiplatelet therapy with aspirin and prasugrel hydrochloride was subsequently started.

**Fig. 4:**
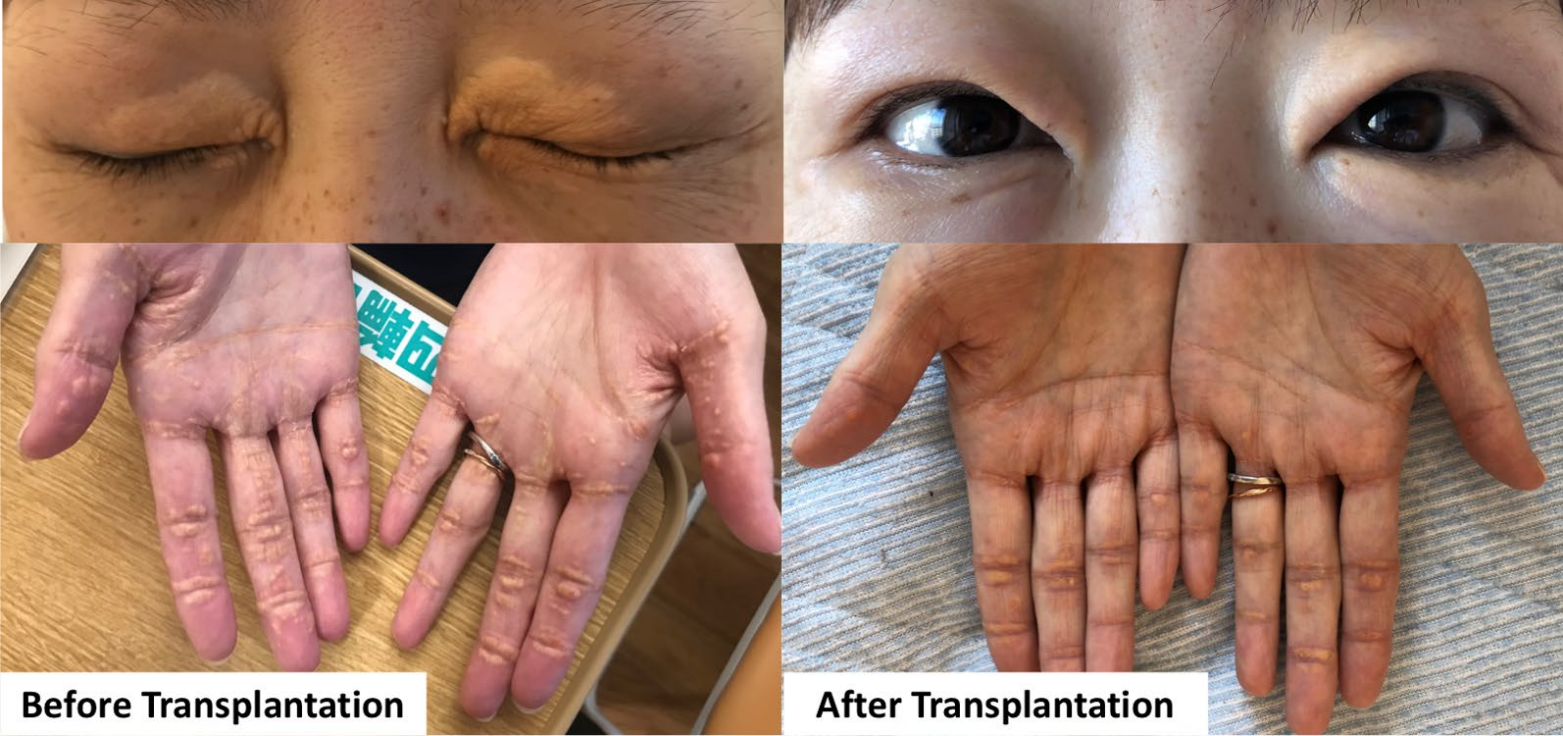
Palpebral and palmar xanthomas before and after transplantation

After the above interventions, her blood pressure recovered, and all the symptoms disappeared. No further elevation of cardiac enzymes, including troponin T and CK-MB, was observed. These values gradually normalized over the course of a month (0.056 ng/mL for troponin T and < 5 U/L for CK-MB on POD 35). The patient was discharged home on POD 46 after treatment for cellular rejection. Serum lipids immediately returned to normal levels (Fig. 1), and the xanthomas had almost disappeared at the 6-month follow-up (Fig. 4). The graft function was good at the 18-month follow-up.

## Discussion

To the best of our knowledge, no previous report has described the potential risk that PBC-related dyslipidemia will lead to ACS after LT.

PBC is closely related to dyslipidemia due to cholestasis, and 75–95% of the patients with PBC have dyslipidemia [6]. Cholestasis reduces bile acid secretion, leading to diminished bile acid synthesis and down-regulation of hepatic cholesterol synthesis. The effectiveness of combination therapy using UDCA and fibrate in improving biliary enzymes and the Mayo Risk Score has been confirmed in retrospective and prospective studies [7–10], and it is the standard treatment today. On the other hand, although dyslipidemia is a common finding in PBC, the risk of cardiovascular events in PBC patients is not higher than that of the general population. The cumulative incidences of ACS in the Asian PBC population are not high [11]. A similar incidence between PBC and normal populations has also been reported in Western countries [2, 12]. These findings suggest that cholesterol metabolism in PBC is different from that in the normal population. Previous studies demonstrated that low lecithin-cholesterol acyltransferase (LCAT) levels and high concentrations of lipoprotein-X and adiponectin concentrations play protective roles against atherogenesis in PBC [12–16]. Particularly, decreased LCAT function leads to changes in lipoprotein composition, decreased LDL and increased lipoprotein-X [15, 16]. Lipoprotein-X prevents LDL oxidation, thus protecting endothelial cells and slowing atherosclerosis [17, 18]. Adiponectin, which is considered to be associated with protection against atherosclerosis, is also increased in patients with PBC compared with controls [14].

While cohort studies are rare regarding this topic, an LT center in the United States reported that the incidence of ACS (including unstable angina and myocardial infarction) after LT was 5.4% among 389 recipients [19]. In addition to traditional coronary risk factors (e.g., hypertension, dyslipidemia), diabetes mellitus, age, pretransplant requirement for vasopressors, and specific pathology, such as nonalcoholic steatohepatitis, are known to increase the risks [19, 20]. In the present patient, we did not see her dyslipidemia as a major problem because she was a young adult without any other risks, and her electrocardiogram and echocardiogram before surgery were normal. However, the refractory dyslipidemia surely did harm. As a reference, the median values of serum total cholesterol and triglyceride before LT in all 125 PBC recipients at Kyoto University were 110 mg/dL (range 25–793) and 100 mg/dL (range 20–469), respectively. She had the 8th highest total cholesterol and 2nd highest triglyceride levels before LT at this institution. Moreover, her peak value of total cholesterol 1 year prior to LDLT was 1287 mg/dL. The long history of PBC and associated severe dyslipidemia for 10 years would have accelerated her atherosclerosis, causing latent stenosis in the coronary artery. Consequently, this inapparent stenosis might have become apparent due to the unstable hemodynamics during the acute phase after LDLT.

**Fig. 5:**
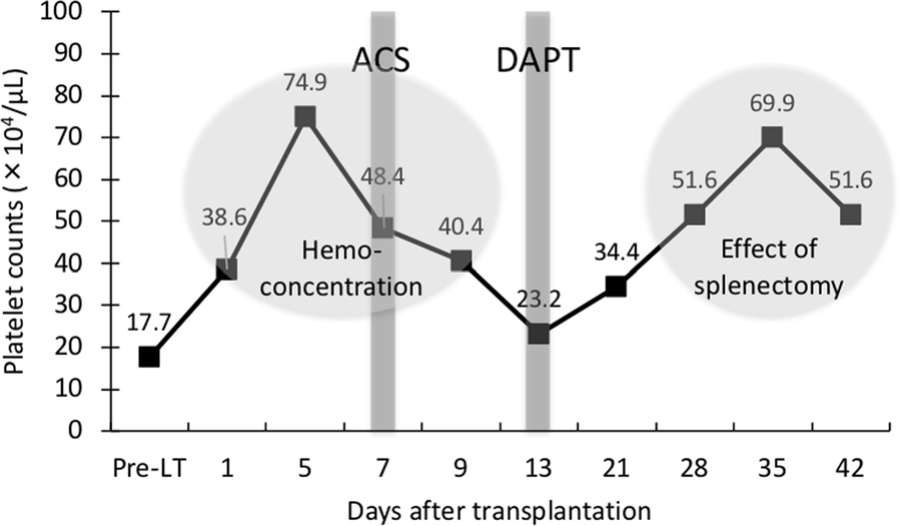
Chronological change in platelet counts. *LT* liver transplantation; ACS, acute coronary syndrome; *DAPT* dual antiplatelet therapy

Hemoconcentration caused by the post-LT dehydration or hypovolemia might potentially contribute to the pathogenesis of ACS as well as atherosclerosis. In the present case, the platelet count reached a peak with 74.9 × 10^4^/μL on POD 5. Generally, the platelet counts in LDLT recipients with splenectomy normally reach a nadir at POD 5, but returns to preoperative levels by day 14, then reaches a peak and levels off after one month [5]. Considering that her pre-LT platelet count was 17.7 × 10^4^/μL, the value on POD 5 was extremely high. Chronological change in platelet counts is shown in Fig. 5. Although we did not recognize, hemoconcentration could have existed early after LT. The increased urea nitrogen (36 mg/dL) and creatinine levels (0.83 mg/dL) and decreased estimated glomerular filtration rate (60.2 mL/min/1.73m^2^) on POD 5 would support our speculation. The association between ACS and essential thrombocythemia has been previously described [21, 22]. Although the increase in the present case was transient, its potential negative influence cannot be denied.

ABO incompatibility and rejection are also well-known risk factors for morbidity after LT [23–25]. However, based on our analyses, no one experienced ACS among 84 ABO-incompatible LTs and 142 recipients with post-LT rejection from 2006 to 2017 [5]. While it has not been studied in the field of LT, a previous report in the field of renal transplantation found no clear association between allograft rejection and ACS among 14.237 patients [26]. Therefore, these two factors seem less relevant to the pathogenesis of ACS.

Although we acknowledge that this is a very rare complication, early postoperative CAD could easily lead to serious morbidity and mortality; thus, the preoperative evaluation of the coronary artery should be considered for selected patients. In particular, a patient with a long history of PBC and drug-resistant dyslipidemia would be a suitable candidate. According to a recent report, the selective use of dobutamine stress echocardiography, CCTA and coronary angiography would be a safe and feasible approach in LT recipients [27]. However, considering the medical cost and burden, widespread evaluation in low-risk patients is not realistic. Through this experience, at Kyoto University, a PBC patient will be referred to a cardiologist when xanthomas, drug-resistant dyslipidemia, or other cardiovascular risk factors (smoking, arterial hypertension, obesity, and diabetes mellitus) exist regardless of age. At the same time, more careful attention to perioperative fluid management is demanded in these patients.

In conclusion, we want to raise the question of whether screening for coronary arteries in PBC patients with dyslipidemia is needed. In a highly invasive procedure such as organ transplantation where failure is unacceptable, a higher level of preoperative screening would be demanded even in young patients if their risk is high.

## Data Availability

The data that support the findings of this study are available from the corresponding author upon reasonable request.

## Abbreviations

ACS: Acute coronary syndrome
CAD: Coronary artery disease
CCTA: Coronary CT angiography
CK-MB: Creatinine kinase MB
CT: Computed tomography
DES: Drug-eluting stent
LDL: Low-density lipoprotein
LDLT: Living donor liver transplantation
LT: Liver transplantation
PBC: Primary biliary cholangitis
UDCA: Ursodeoxycholic acid
